# Oral health effects of nicotine pouches in snus users: clinical and immunological findings

**DOI:** 10.1007/s00784-026-07087-0

**Published:** 2026-08-22

**Authors:** Sara Alizadehgharib, Anna-Karin Östberg, Anna Lehrkinder, Jisha Varghese, Taha Abdul Jabar, Filip Ilver, Peter Lingström

**Affiliations:** 1 Department of Oral Microbiology and Immunology, Institute of Odontology, Sahlgrenska Academy, University of Gothenburg, Box 450, Gothenburg, 405 30 Sweden; 2 Department of Cariology, Institute of Odontology, Sahlgrenska Academy, University of Gothenburg, Gothenburg, Sweden; 3 Public Dental Service (Folktandvården Breared and Tvååker), Region Halland, Sweden

**Keywords:** Cytokines, Inflammation, Oral mucosa, Clinical trial, Nicotine pouches

## Abstract

**Objectives:**

Changes in the oral mucosa are characteristic of snus-induced lesions (SILs), typically occurring at the site of pouch placement. These lesions are reversible after cessation. This study evaluated the effects of two non-tobacco-based nicotine pouches (ZYN^®^ Dry and ZYN^®^ Moist) on SIL severity, salivary cytokines, epithelial thickness, and inflammatory cell presence after 28 days of exclusive use.

**Materials and methods:**

In this open-label, randomized longitudinal study, 45 daily snus users were assigned to either the dry formulation (*n* = 23) or the moist formulation (*n* = 22) for four weeks. Clinical assessments were performed at baseline, day 14, and day 28. Lesions were graded on a four-point scale. Biopsies and saliva samples were collected at baseline and day 28.

**Results:**

Both groups showed significant improvement in lesion severity (*p* < 0.0001). No significant changes were observed in epithelial thickness, inflammatory cell infiltration, total protein levels, IL-1β, TNF-α, or MCP-1. IL-8 levels increased significantly in the dry formulation group (*p* < 0.01).

**Conclusions:**

Switching to nicotine pouches improved SILs without major changes in the inflammatory markers assessed.

**Clinical relevance:**

Nicotine pouches were associated with improvement of snus-induced lesions in this study population.

**Trial registration:**

ISRCTN13243849, registered 01/09/2022.

**Supplementary Information:**

The online version contains supplementary material available at https://doi.org/10.1007/s00784-026-07087-0.

## Introduction

Swedish snus is a widely used smokeless tobacco product and has been used as an alternative to combustible cigarettes. However, adverse oral effects, including gingival hyperplasia and snus-induced lesions (SILs), are commonly observed among snus users [1]. Tobacco products have also been associated with effects on periodontal tissues, including the cementum, alveolar bone, and periodontal ligament, as well as with periodontal disease [2].

In recent years, the use of non-tobacco-based nicotine pouches (NPs) has increased substantially. These products are available in different nicotine strengths, flavors, and formulations. The present study investigated two commercially available nicotine pouch products, ZYN^®^ Dry (dry formulation) and ZYN^®^ Moist (moist formulation), which differ in moisture content and pouch composition. Unlike conventional snus, nicotine pouches do not contain tobacco leaf and therefore lack several tobacco-specific harmful constituents, such as nitrosamines and polycyclic aromatic hydrocarbons [3].

Previous studies have demonstrated differences in nicotine release and uptake between conventional snus and nicotine pouch products, with considerable inter-individual variability reported [4–7].

Given the established effects of snus on the oral mucosa, concerns have been raised regarding whether nicotine pouches may induce similar oral changes. A previous clinical study reported that substitution of regular snus with a dry nicotine pouch product was associated with improvement of pre-existing snus-induced lesions [8]. However, limited information is available regarding the effects of nicotine pouches on the oral environment, particularly with respect to histopathological findings, inflammatory responses, and salivary biomarkers.

Therefore, the primary objective of the present study was to evaluate the effects of two non-tobacco-based nicotine pouch formulations (dry and moist) on the severity of pre-existing oral mucosal lesions following four weeks of exclusive use. Clinical, histopathological, and immunological assessments were performed at baseline and after 28 days of product substitution.

## Materials and methods

This was an open-label, two-armed, randomized, longitudinal study designed to assess the effects on various oral variables in daily tobacco-based snus users who completely substituted their snus with either the dry or moist non-tobacco-based nicotine pouch formulations. The primary endpoint was the change in lesion severity from baseline to day 28. The investigational products (IPs) consisted of unflavored (Smooth) or flavored (Cool Mint and Citrus) NPs with different nicotine strengths (dry 6 mg and moist 9 mg). The difference in nicotine content was compensated for by the higher nicotine extraction from the dry formulation compared with the moist formulation, resulting in an overall comparable nicotine delivery profile. According to previously published chemical analyses [3], the dry nicotine pouch formulation contained fillers, a stabilizer, pH adjusters, nicotine salt, flavorings, and a sweetener. The moist formulation additionally contained water, glycerol, and sodium chloride.

The study was monitored by Clinical Trial Consultants AB (CTC), which systematically and independently reviewed all study-related activities and documentation to verify that they were conducted, and that data was recorded, analyzed, and reported, in accordance with the study protocol and other applicable regulatory requirements.

### Risk and benefit assessment

All participants were daily snus users for at least one year, familiar with the effects of nicotine, and at no risk of developing new dependence. Potential adverse effects were expected to be minor based on prior clinical trials [9–12], and individuals belonging to any risk groups, as defined by the exclusion criteria, were excluded. Nicotine exposure did not exceed participants’ usual intake, minimizing acute risks. No serious adverse events (AEs) have been reported in previous studies, aside from typical nicotine-related effects such as salivation, nausea, and dyspepsia.

As in a standard Phase I study, there was no direct benefit for participants, aside from an oral examination. Participant safety and well-being were prioritized.

### Ethical conduct of the study

The study and the clinical study protocol were approved by the Swedish Ethical Review Authority (approval number 2022-03919-01).

The study was conducted in accordance with ethical principles that have their origin in the Declaration of Helsinki and in compliance with the International Conference of Harmonization (ICH) of Technical Requirements for Pharmaceuticals for Human Use E6(R2) guideline for Good Clinical Practice guidance, the EU Clinical Trials Directive, and applicable local regulatory requirements. The study was registered in the International Standard Randomized Controlled Trial Number (ISRCTN) registry (ISRCTN13243849).

### Recruitment

Participants were recruited from CTC’s database of healthy volunteers and through strategic marketing campaigns. Advertisements on social media and other media channels (e.g., newspapers, internet, radio), and local distribution of flyers were used to reach the target audience. All advertising texts were approved by the independent ethics committee.

### Participant information and consent

All participants were given adequate verbal and written information before any study-specific assessments were performed. Participants were given the opportunity to ask questions about the study and sufficient time to consider participation before signing the informed consent form (ICF). A copy of the participant information, including the signed ICF, was provided to each participant.

### Method of assigning subjects to product test groups

At Visit 3, participants were randomized to one of two treatment arms: a dry nicotine pouch formulation (ZYN^®^ Dry) or a moist nicotine pouch formulation (ZYN^®^ Moist). Within each arm, participants could select and change flavor as desired. Randomization was computer-generated using SAS Proc Plan (Version 9.4; SAS Institute Inc., Cary, NC, USA), and the randomization list, including subject IDs and treatment allocations, was prepared by CTC.

### Blinding

This was an open-label study; therefore, no formal blinding procedures were implemented.

### Number of participants

The study included 45 randomized participants, with the aim of obtaining 40 fully evaluable participants. In total, 59 subjects were screened, 45 were randomized, and all 45 completed the study, i.e., they used the IPs ad libitum for 4 weeks and attended all study visits. Of the randomized participants, 16 were female (36%) and 29 were male (64%). Specifically, 23 participants (13 male and 10 female) were randomized to the dry formulation, and 22 participants (16 male and 6 female) were randomized to the moist formulation (Table 1). The study was conducted between 22 November 2022 and 2 May 2023. Participant flow throughout the study is presented in Supplementary Figure S1.

In total, 14 participants were withdrawn prior to randomization at Visit 3. There were four screening failures, three participants withdrew consent for personal reasons, and five participants were designated as reserves. Two participants were lost to follow-up prior to randomization after failing to attend consecutive scheduled visits and could not be reached despite multiple contact attempts.

**Table 1:** Summarized participant disposition, with details on screening failures. A total of 59 subjects were screened, with 45 randomized into two treatment arms: 23 to the dry formulation (ZYN Dry) and 22 to the moist formulation (ZYN Moist). Fourteen subjects were withdrawn prior to randomization at visit 3, including 4 screening failures, 3 withdrawals of consent, 5 reserves, and 2 lost to follow-up (“Other”)

	Total (n = 59)
Screened subjects	59
Withdrawn prior to dose	14
Reason for withdrawal prior to dose	
Screening Failure	4
Withdrawal Of Consent	3
Reserve	5
Other	2
Subjects included in study	45
Randomized to arm	
ZYN Dry	23
ZYN Moist	22
Withdrawn subjects	0
Completed subjects	45
Included in Full analysis set	45
Subjects at each visit	
Visit 1 (screening)	59
Visit 2	46
Visit 3	45
Visit 4	45
Visit 5	45

### Inclusion criteria

To be included in the study, participants had to meet the following criteria: be healthy males or females aged 21–55 years and be willing and able to provide written informed consent. Participants were required to have used tobacco-based snus for ≥ 1 year, with a minimum weekly consumption of three cans, and to obtain ≥ 90% of their total nicotine intake from tobacco-based snus. They also had to be non-smokers (defined as having smoked no more than five packs in total ever and none during the past year). In addition, participants were required to have normal stimulated salivary secretion rate (> 0.7 mL/min), at least 24 natural teeth, and overall good oral health, as assessed by the investigator.

Female participants of childbearing potential were required to use an adequate contraceptive method for the duration of the study. Sexual abstinence was permitted if it was the participant´s usual and preferred lifestyle.

### Exclusion criteria

Participants were excluded if they had a history of diagnosed hypertension or any cardiovascular disease. Any surgical or medical condition, including abnormal salivation (also pharmaceutically induced), or a history of such conditions that, in the investigator´s judgment, could interfere with the absorption, distribution, metabolism, or excretion of the investigated product (IP), pose a risk to the participant, influence the study results, or impair participation also resulted in exclusion. Participants who were pregnant, breastfeeding, or intending to become pregnant during the study were excluded. Additional exclusion criteria included a history of severe allergy/hypersensitivity, or ongoing manifestations thereof, to aroma compounds (including fragrances and/or flavorings), as assessed by the investigator. Participants with severe oral conditions such as open caries lesions, severe periodontal disease, soft-tissue lesions (other than gingival hyperplasia related to snus use), or extensive prosthetic work (e.g., multiple implants, partial dentures, or dental veneers) were also excluded. Further exclusion criteria included current or past alcohol abuse and/or the use of anabolic steroids or drugs of abuse, as judged by the investigator. Use of antibiotics within 4 weeks prior to screening was prohibited. Participants who intended to change their nicotine consumption habit, including plans to stop using nicotine products, within 4 months of the screening visit were also excluded, as judged by the investigator. Finally, participants undergoing other dental treatments during the study period were excluded.

### Study design

Participants attended a screening visit followed by four treatment visits on separate days. All tests and screenings were carried out at the research laboratory at the Department of Cariology, Institute of Odontology, University of Gothenburg. The screening visit (Visit 1) took place within 5 weeks prior to the first treatment visit and included an eligibility assessment, including evaluations of smoking and oral tobacco/nicotine use, a brief oral examination, urine pregnancy testing (for women of child-bearing potential only), and collection of medical history. Salivary secretion rate and buffer capacity were also assessed. At the end of the screening, participants were asked about their flavor preferences for the IPs.

During the first treatment visit (Visit 2, Day − 28), participants attended the clinic for a routine dental examination, including assessment of oral mucosal lesions. They electronically recorded the number of tobacco-based snus pouches used per day, the timepoints of the first and last pouch of the day, and the estimated duration of use per pouch over a 4-week period (up to Visit 3, day 0).

At the second treatment visit (Visit 3, day 0), a routine dental examination, including assessment of oral mucosal lesions, was performed, and saliva was collected for cytokine analysis. Visit 3 was selected as the baseline assessment because it represented the final evaluation immediately prior to substitution of tobacco-based snus with nicotine pouches and served as the predefined reference point for all subsequent treatment comparisons. Participants continued using their usual tobacco-based snus products between Visit 2 and Visit 3, and lesion severity appeared similar at both assessments. Prior to the assessment of oral mucosal lesions, clinical photographs were taken. Biopsies were obtained from the upper vestibular mucosa corresponding to the participant’s habitual pouch placement site. At Visit 5, efforts were made to collect the biopsy from a comparable anatomical area based on the clinical appearance and location of the lesion. However, minor variations in biopsy location between visits could not be completely excluded. Participants were then randomized to substitute their tobacco-based snus with either the dry formulation or the moist formulation for 4 weeks. They were provided with the IPs and instructed to use them ad libitum, according to their usual pattern of use. Participants were also instructed to electronically record the number of nicotine pouches used per day, the timepoints of the first and last pouch of the day, and the estimated duration of use per pouch for 4 weeks (until Visit 5, day 28).

At the third treatment visit (Visit 4, day 14), a routine dental examination was performed, and clinical photographs of the oral lesions were taken. Additional IPs were provided if needed, and participants could change flavor upon request.

During the fourth treatment visit (Visit 5, day 28), a routine dental examination (including assessment of oral mucosal lesions) was performed, and saliva was collected for cytokine analysis. A biopsy of the oral mucosa was also obtained from the representative area.

AEs were collected through participant interviews from the start of substitution of tobacco-based snus with nicotine pouches (Visit 3) until the final treatment visit (Visit 5).

### General restrictions

Participants were required to fully replace their tobacco-based snus products with nicotine pouches for 4 weeks, starting from the second treatment visit (Visit 3), and to refrain from using any other nicotine products until the end of the study (Visit 5). Participation in other clinical studies or the use of antibiotics were also prohibited during this period. Subjects were instructed to refrain from approximal tooth cleaning for 48 h and toothbrushing for 24 h prior to each treatment visit (Visit 2–5). They were also required to use their regular toothbrush (electrical or manual) and a standard fluoride-containing toothpaste (sodium fluoride; 1450 ppm F) from Visit 2 to Visit 5.

### Oral mucosal lesions

Oral mucosal lesions were assessed and graded from 1 to 4 according to Axéll et al. [13]. Clinical photographs were taken at Visits 3 and 5, during which participants were instructed to lift their upper lip with their fingers. The grading of the oral mucosal lesions was categorized as follows: Degree 1 – a superficial lesion matching the color of the surrounding mucosa with slight wrinkling but no visible thickening; Degree 2 – a superficial lesion that is whitish or yellowish with wrinkling but no visible thickening; Degree 3 – a wrinkled lesion ranging from whitish-yellowish to brown, with normal-colored furrows and obvious thickening; and Degree 4 – a heavily wrinkled lesion from white-yellowish to brown with deep reddened furrows and/or significant thickening.

### Histopathological evaluation

Punch biopsies (3 mm in diameter) were obtained under local anesthesia, following the routine clinical biopsy procedures at Visits 3 and 5. The procedure was not associated with any major discomfort or significant AEs.

Biopsy samples were prepared by Histocenter AB, Mölndal, Sweden. They were fixed in 4% formaldehyde, processed, mounted on glass slides, and stained with Hematoxylin and Eosin. Epithelial thickness and the presence of total inflammatory cells were analyzed using computer software (Biopix iQ 3.2.0). Epithelial thickness was measured in micrometers (µm) at the thickest and thinnest regions, excluding sample edges and papilla, and the mean thickness was calculated from these measurements. Cells were quantified based on color intensity and morphology, and results were expressed as the percentage of positively stained cells per selected tissue area. Cell counting was performed in a blinded manner by one observer after calibration with another observer.

### Measurement of total protein concentration in saliva

Total protein concentration was determined using the Pierce™ Bicinchoninic Acid (BCA) Protein Assay Kit (Thermo Fisher Scientific, Gothenburg, Sweden). Reagents A and B supplied with the kit were used to prepare the working reagent. Bovine serum albumin (BSA) served as the protein standard, and serial dilutions were prepared to generate a standard curve ranging from 25 to 2000 µg/mL.

In brief, the BCA working reagent was prepared by mixing 50 parts of Reagent A with 1 part of Reagent B. For each sample and standard, 25 µL was pipetted into a 96-well microplate, followed by the addition of 200 µL of the working reagent. The plate was incubated at 37 °C for 30 min. Absorbance was measured at 562 nm using a microplate reader, and the protein concentrations were calculated by comparing sample absorbance values to the BSA standard curve.

### Measurement of cytokines in saliva

Saliva (1 ml) was collected at Visits 3 and 5. To each sample, 25 µl of protease inhibitor (Sigma, Town, Country) was added, and the samples were stored at -80 °C. Before analysis, the saliva samples were centrifuged for 7 min at 1700 x g, and the supernatants were transferred to new Eppendorf tubes.

Cytokine concentrations in saliva were measured using a Multiplex immunoassay (Bio-Plex Pro Human Cytokine Screening Panel, 48-Plex; Bio-Rad Laboratories, Hemel Hempstead, United Kingdom) according to the manufacturer’s instructions. Briefly, color-coded beads coupled to antibodies specific for each target biomarker were used. Following sample addition, the antibodies on the beads bound to the corresponding biomarkers. Unbound proteins were removed through detergent washes, after which a biotinylated detection antibody was added to form a sandwich complex. A streptavidin-phycoerythrin conjugate was then introduced to bind the biotinylated antibody, completing the detection complex. Samples were analyzed on a Bio-Plex 200 instrument using BioManager analysis software (BioRad, Hercules, CA). Results were reported as PPM.

### Statistics

Statistical comparisons of paired samples were performed using the Wilcoxon matched-pairs signed-rank test. A significance level of *p* < 0.05 was considered statistically significant. GraphPad Prism (GraphPad Software, San Diego, CA, USA) was used for data analysis and figure generation.

No formal a priori power calculation was performed due to limited prior data for reliable effect size estimation. To enhance transparency, effect sizes (r) were calculated for the primary outcome based on the Wilcoxon signed-rank test (r = Z/√N), providing an estimate of the magnitude of the observed effects.

## Results

### Degree of oral mucosal lesions

**Fig. 1:**
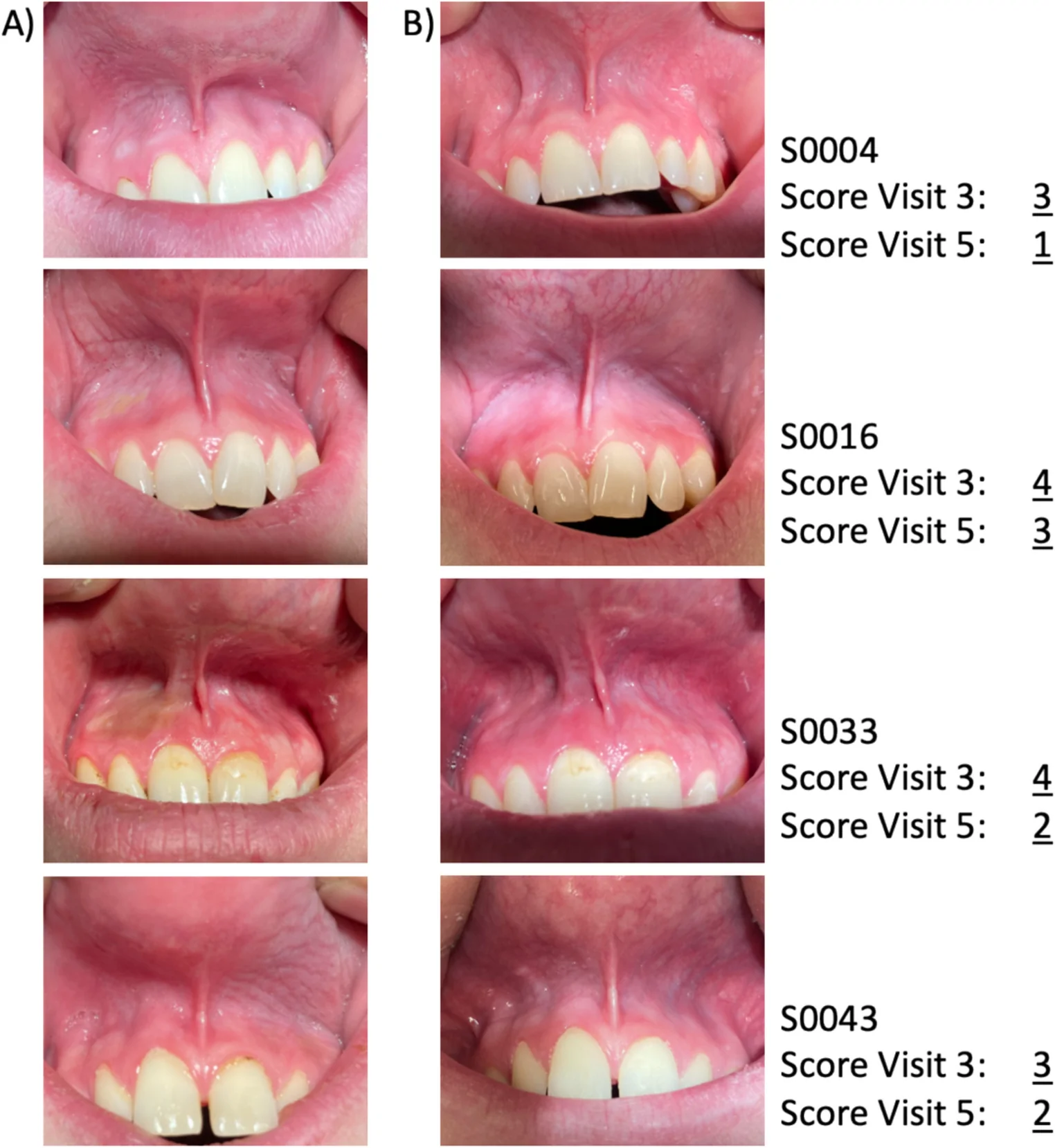
Oral mucosal lesions before (**A**) and after (**B**) switching to non-tobacco-based nicotine pouches. Clinical photographs of four participants taken during visit 3 (while using regular snus) and visit 5 (after exclusively using nicotine pouches for four weeks). The oral mucosal lesions at the pouch placement site were assessed using a four-point clinical scale. *n* = 45 participants

Oral mucosal lesions were assessed, and clinical photographs were taken at each treatment visit (Fig. 1). Normal mucosa, with no observable alterations, was assigned a score of 0. Oral mucosal lesions were graded from 1 to 4 according to Axéll et al. [13]. During Visits 2 and 3, when participants were using tobacco-based snus, 30–50% had oral mucosal lesions graded as 4. No statistically significant difference in lesion severity was observed between Visit 2 and Visit 3 during continued snus use (*p* = 0.2869), supporting the use of Visit 3 as the baseline before nicotine pouch substitution. Following exclusive use of either the dry or moist formulation, noticeable reductions in the proportion of participants with lesions scored as 4 were observed at Visit 4 (14 days) and Visit 5 (28 days). For the dry formulation, the proportions decreased to 17% and 13%, respectively, and for the moist formulation to 36% and 14%, respectively. Additionally, at Visits 4 and 5, an increase in the proportion of participants with oral mucosal lesions scores of 0 or 1 was observed, particularly among those using the dry formulation, increasing from 8.7% at Visit 3 to 13% at Visit 4 and 34.3% at Visit 5.

**Fig. 2:**
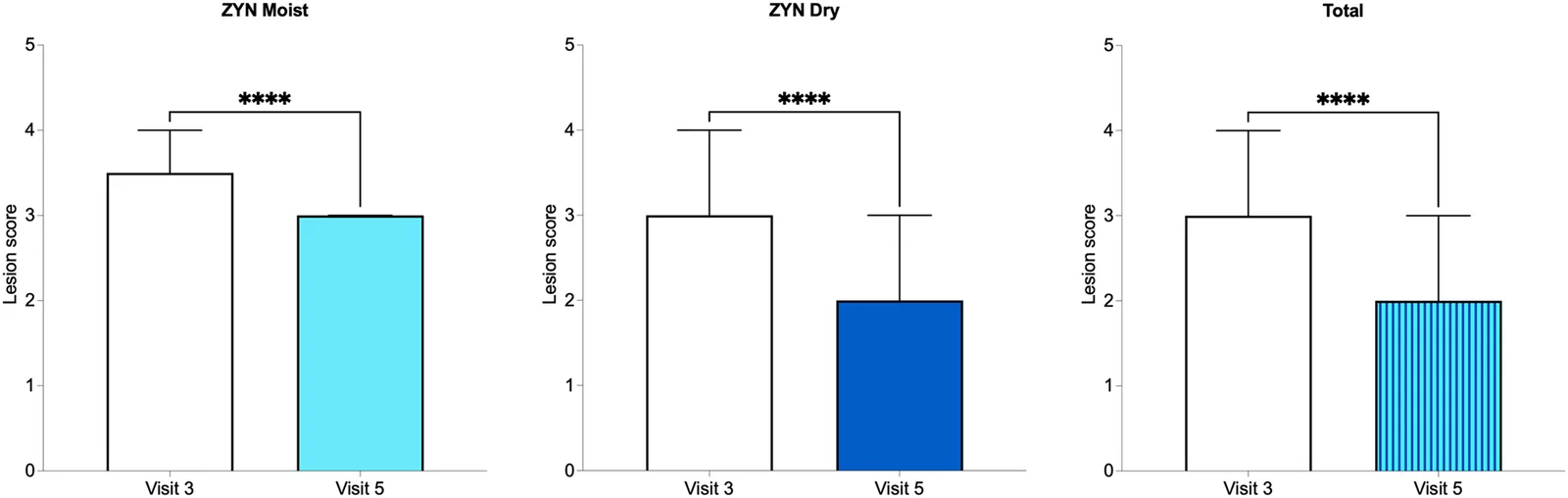
Comparison of mucosal lesion severity at pouch placement site. The severity of mucosal lesions at the pouch placement site was assessed at visit 3 and visit 5 using a four-point clinical scale by Axéll et al. Statistical analysis was performed with Wilcoxon matched-pairs signed-rank test. **** *p* < 0.0001, ** *p* < 0.01. *n* = 45 participants

Both groups showed a significant reduction in lesion severity between Visit 3 and Visit 5 (*p* < 0.0001), with a large effect size (*r* ≈ 0.60). In the moist formulation group, lesion grade decreased significantly from Visit 3 to Visit 5 (*p* < 0.0001). The dry formulation group demonstrated a similar significant improvement over the same period (*p* < 0.0001) (Fig. 2).

### Epithelial thickness and presence of inflammatory cells

Following microscopic evaluation, some samples were excluded due to alignment issues that could result in inaccurate measurements. Specifically, samples were excluded if papillary structures were not clearly visible or if no tissue was present directly adjacent to the epithelial layer. Only slides that were properly embedded, correctly sectioned, and exhibited continuous tissue in direct contact with the epithelium were included in the epithelial thickness analysis. This approach ensured that only precise and reliable data were included in the final evaluation.

**Fig. 3:**
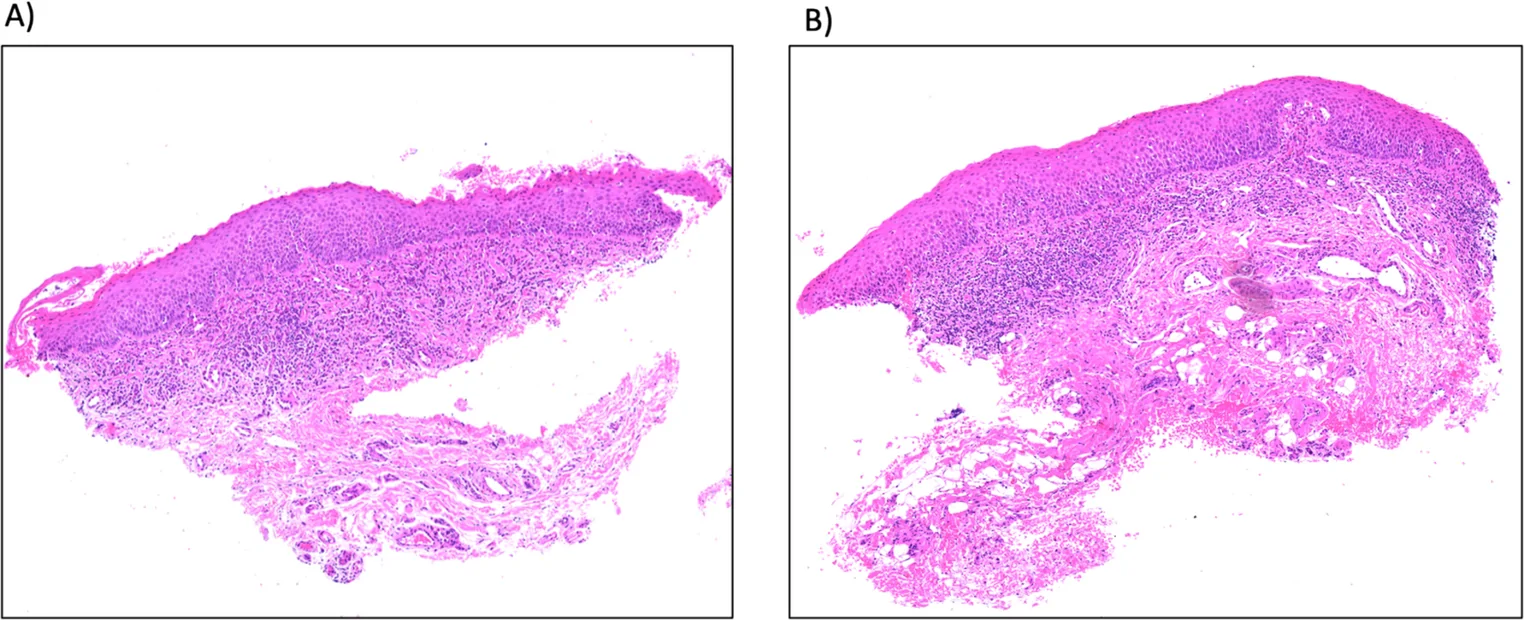
Inflammatory cell presence and epithelial thickness. Representative histological images of snus-induced lesions at visit 3 (**A**) and visit 5 (**B**), stained with hematoxylin and eosin, illustrating inflammatory cell infiltration and epithelial thickness

**Fig. 4:**
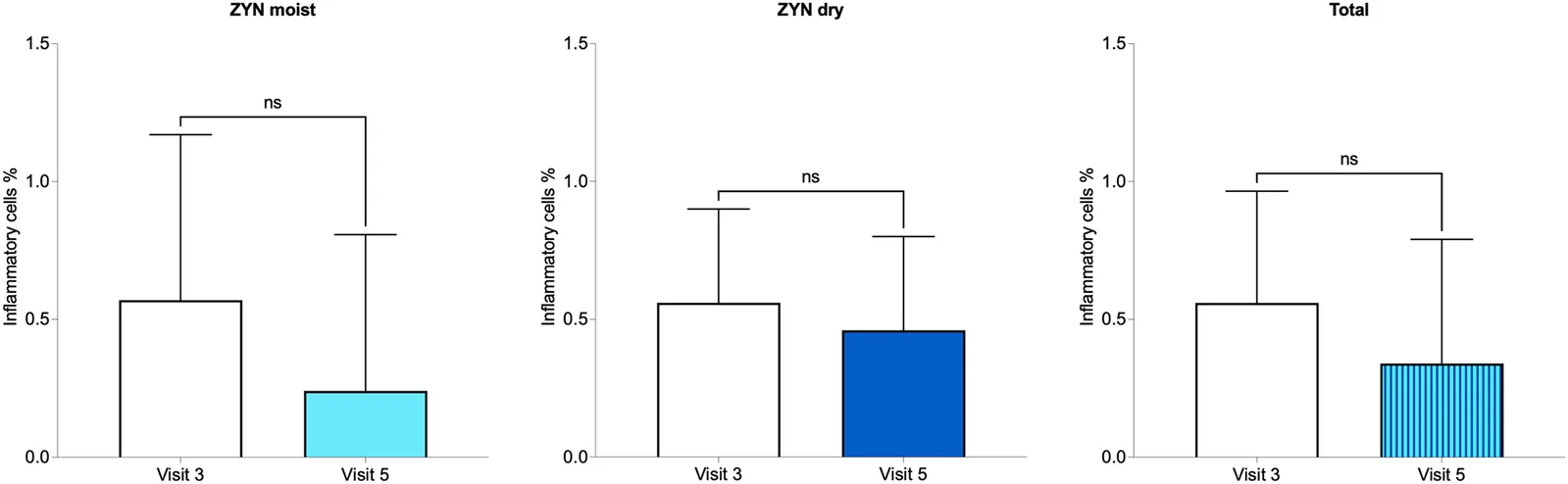
Inflammatory cell presence in snus-induced lesions. The presence of inflammatory cells in biopsies from snus-induced lesions at visit 3 and visit 5 was quantified as the percentage of positively stained cells per selected tissue area. Statistical analysis was conducted using the Wilcoxon matched-pairs signed-rank test. The notation ‘ns’ indicates no statistically significant differences. *n* = 45 participants

**Fig. 5:**
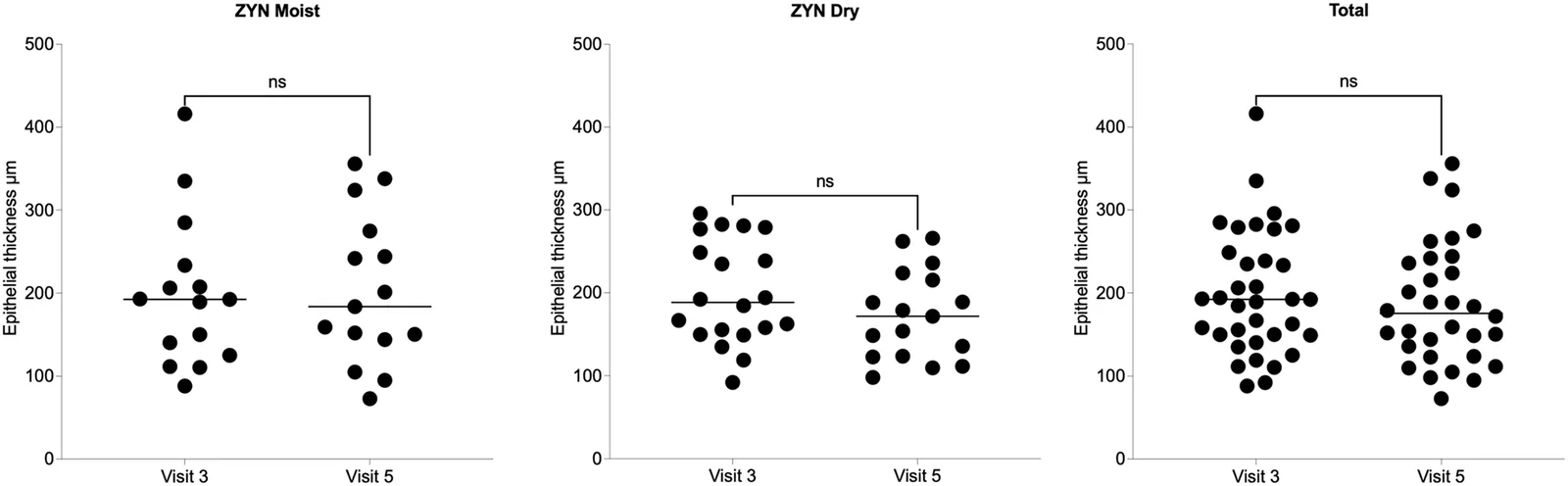
Epithelial thickness in snus-induced lesions at visit 3 and visit 5. Epithelial thickness (µm) was measured in biopsies from snus-induced lesions at visit 3 and visit 5. Statistical comparisons between visits were made using the Wilcoxon matched-pairs signed-rank test to assess changes in epithelial thickness over time. The notation ‘ns’ indicates no statistically significant differences. *n* = 45 participants

The thickness of the epithelial layer and the percentage of inflammatory cells were assessed in oral mucosal biopsies collected at Visit 3 and Visit 5 (Fig. 3). The analysis showed that neither the percentage of inflammatory cells (Fig. 4) nor the epithelial thickness (Fig. 5) changed significantly over this period in either the dry or moist formulation groups.

### Measurement of total protein concentration in saliva

**Fig. 6:**
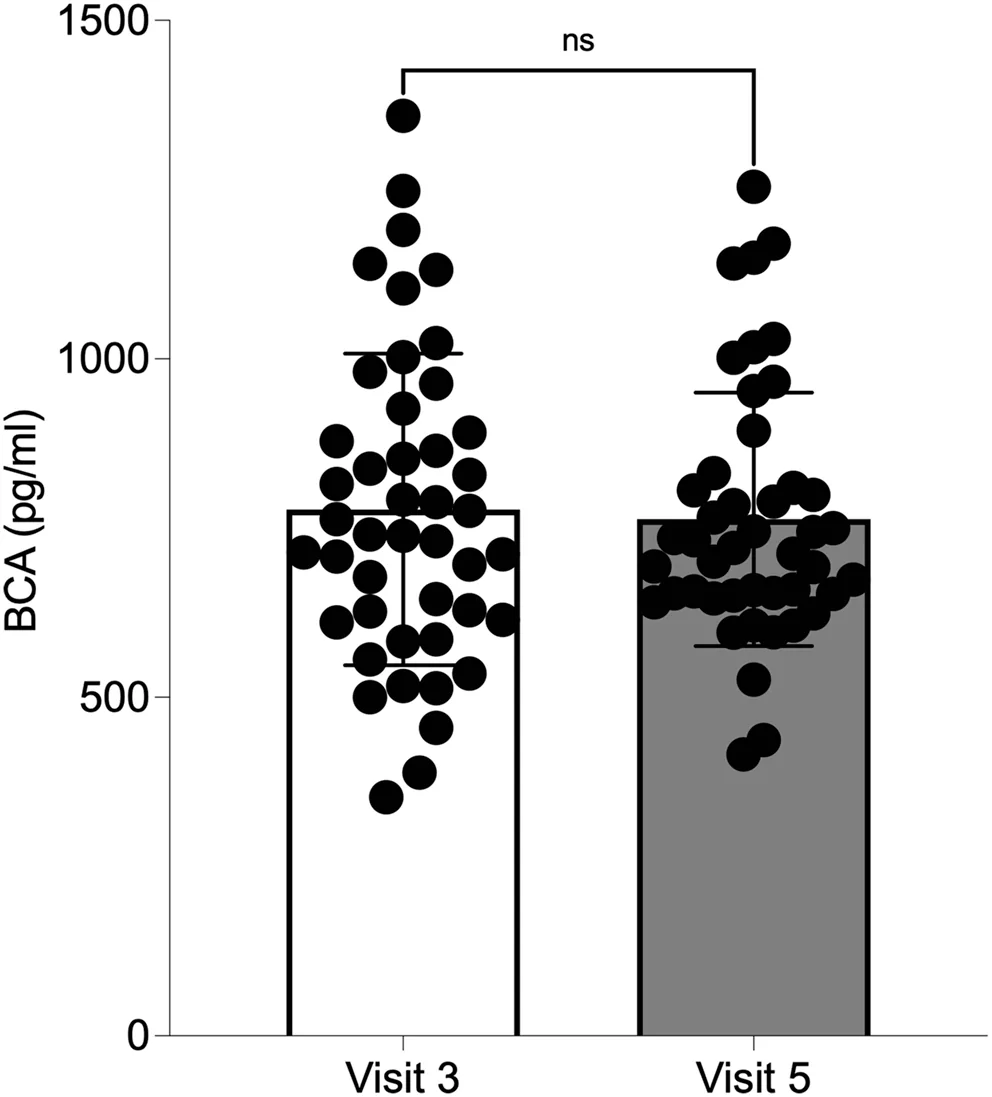
Total protein levels in saliva. Total protein levels were measured in saliva samples collected at visit 3 and visit 5. Statistical analysis was performed using the Wilcoxon matched-pairs signed-rank test to evaluate changes in protein concentration between visits. The notation ‘ns’ indicates no statistically significant differences. *n* = 45 participants

Total protein concentration in saliva was measured, and no statistically significant differences were observed between Visit 3 and Visit 5 in either group (Fig. 6).

### Measurement of cytokines in saliva

**Fig. 7:**
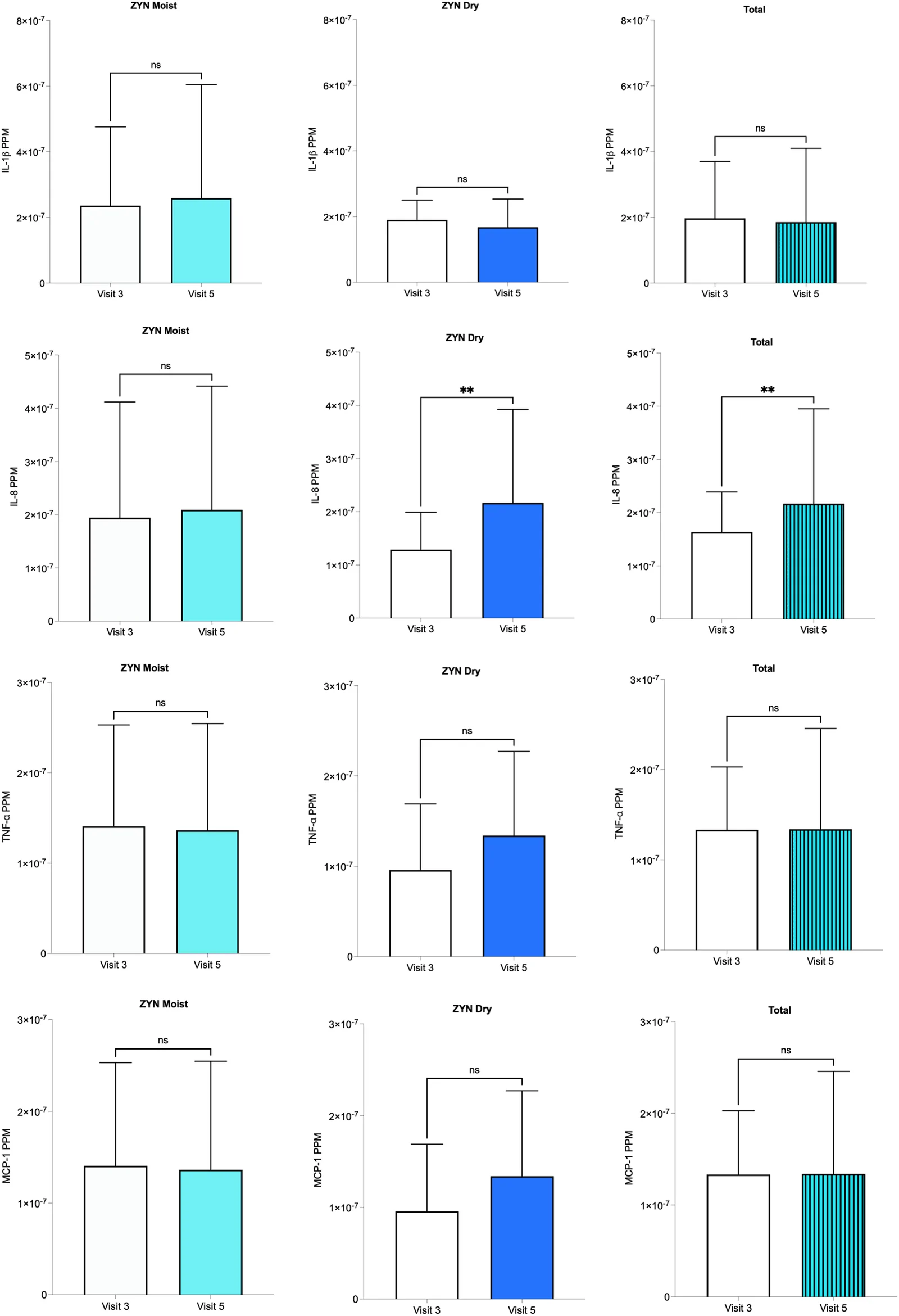
Cytokine levels in saliva. Cytokine levels in saliva samples collected at visit 3 and visit 5 were measured. The concentrations of pro-inflammatory cytokines IL-1β, IL-8, TNF-α, and MCP-1 were quantified using a multiplexed bead-based immunoassay. Cytokine concentrations were normalized to total protein levels. Statistical analysis was conducted using the Wilcoxon matched-pairs signed-rank test. The notation ‘ns’ indicates no statistically significant differences. ** *p* < 0.01. *n* = 45 participants

Cytokine concentrations in saliva were measured using a multiplex assay. Only mean cytokine levels within the detectable range of the standard curve are presented. Values below the lower limit of the standard curve were assigned the lowest standard value, while values above the upper limit were assigned the highest standard value. Cytokine concentrations were normalized to total protein concentration, and the results are shown in Fig. 7. No significant differences were observed in the levels of the pro-inflammatory cytokines interleukin-1 beta (IL-1β), tumor necrosis factor alpha (TNF-α), and the chemokine monocyte chemoattractant protein-1 (MCP-1) in saliva samples between Visit 3 and Visit 5. In contrast, levels of the chemokine interleukin-8 beta (IL-8) were significantly increased (*p* < 0.01) from Visit 3 to Visit 5, but only in the dry formulation group.

### Analysis of pattern of use

In the dry formulation group, the cumulative exposure time was 10.4 h at Visit 3 and decreased slightly to 10.1 h at Visit 5. In the moist formulation group, the cumulative exposure time was 10.7 h at Visit 3 and remained essentially unchanged at 10.6 h at Visit 5.

### Safety and tolerability

*Ad libitum* use of dry formulation NPs and moist formulation NPs was found to be safe and well tolerated among current daily users of tobacco-based snus in this study. No deaths, serious adverse events, or withdrawals due to AEs were reported. Two participants (4.4%) reported a total of two AEs, both cases of the common cold. Both AEs were assessed as mild and unlikely to be related to the IPs, and they resolved within 4–5 days. No additional AEs were reported, and no differences in AE frequency were observed between the dry and moist formulation groups.

## Discussion

The aim of this study was to evaluate oral mucosal lesions in terms of clinical presence, visual grading, and histopathological characteristics following replacement of tobacco-based snus with either dry or moist nicotine pouch formulations over a four-week period. A secondary aim was to compare salivary protein expression after substitution with these products over the same duration. The findings indicate that replacing snus with non-tobacco-based nicotine pouches led to gradual improvement of pre-existing oral mucosal lesions in healthy snus users during the study period. However, despite these clinical improvements, the histological characteristics of these lesions, including epithelial thickness and inflammatory cell presence, remained unchanged. Furthermore, no statistically significant differences were observed in total protein levels or in MCP-1, IL-1β, and TNF-α expression in saliva. However, IL-8 levels were significantly increased in the dry formulation group.

The use of NPs has increased markedly in recent years [14, 15]. Because NPs are neither combusted nor derived from tobacco leaf, they could offer a lower-risk alternative to traditional nicotine products [16]. However, as they are relatively new to the market, scientific evidence regarding their effects on the oral environment remains limited. In particular, further research is needed to clarify their impact on oral immune responses, saliva composition, the histological health of oral soft tissues.

Oral mucosal lesions associated with smokeless tobacco (snus) have previously been documented at the pouch placement site [1, 13]. These lesions are generally reversible within weeks after cessation and do not appear to be premalignant [17]. Although the underlying mechanisms are not fully understood, local chemical and mechanical irritation caused by snus constituents has been proposed to contribute to these mucosal changes [18, 19]. The oral mucosa is susceptible to chemical and mechanical irritation because of its permeability and vascularization [20, 21]. Such irritation may contribute to local inflammatory responses in the oral mucosa. A previous study by Axéll et al. demonstrated histological inflammation at lesion sites in snus users [13]. Inflammation involves various white blood cells that can release mediators such as cytokines, which contribute to local immune and inflammatory processes.

In the present study, replacement of snus with non-tobacco-based nicotine pouches resulted in a clear reduction in lesion severity in both groups. Lesion severity at Visit 5 was significantly reduced in both the dry and moist formulation groups compared with Visit 3. These findings are consistent with those of a previous study reporting improvement of pre-existing mucosal lesions following substitution of regular snus with non-tobacco-based nicotine pouches [8].

A correlation has previously been suggested between the clinical grading of snus-induced lesions and corresponding histologic changes, including histological inflammation in clinically severe lesions [22]. Building on these findings, we examined the presence of inflammatory cells in biopsies collected from lesion sites at Visit 3, when participants were still using tobacco-based snus, and at Visit 5, after four weeks of non-tobacco-based NP use. We also assessed epithelial thickness, as changes in thickness may reflect tissue responses to chronic irritation or inflammation [23]. The results showed no significant differences in the percentage of inflammatory cells or in epithelial thickness between Visit 3 and Visit 5 in either the dry formulation or the moist formulation groups. However, although not statistically significant, a slight reduction in inflammatory cells was observed in the moist formulation group at Visit 5.

Given the diverse range of cell types present in the oral cavity, including those in the pulp, gingival crevicular fluid, and epithelium [24, 25], many of these cells may come into contact with substances released from snus, potentially triggering inflammation, as previously suggested by Axéll et al. [13]. Cytokines play an important role in inflammatory regulation and salivary cytokine levels have previously been associated with inflammatory oral conditions, including gingivitis, periodontitis, and oral lesions [26–29]. Elevated levels of IL-1, IL-6, IL-8, and TNF-α have also been reported in the saliva of snus users compared with non-tobacco-based controls [30]. In the present study, no significant differences were observed in total protein levels or in the pro-inflammatory cytokines IL-1β, TNF-α, or MCP-1 between Visit 3 and Visit 5. However, IL-8 levels were significantly increased in the dry formulation group. This finding was unexpected, as inflammatory cytokine levels were hypothesized to decrease following the observed clinical improvement in lesion severity. IL-8 has previously been implicated in inflammatory and tissue repair processes [31]. However, the biological significance of the observed increase remains unclear and should therefore be interpreted cautiously. In addition, a potential contribution of nicotine itself to the observed IL-8 response cannot be excluded.

Within the limitations of the present study, substitution of regular snus with non-tobacco-based nicotine pouches was associated with improvement of pre-existing snus-induced oral lesions. However, it cannot be excluded that the observed improvements may partly reflect reduced exposure to tobacco constituents or spontaneous lesion regression over time. Although the study was designed as a longitudinal within-subject comparison, the absence of a continued snus-use control group or a nicotine-abstinent control group limits the ability to draw definitive conclusions regarding causality. In addition, participants were allowed to select among available flavours and could change flavour during the intervention period. Although this approach reflected real-world product use, it cannot be excluded that flavouring agents may have influenced oral mucosal responses independently of the nicotine pouch formulation itself. The present study was not designed to evaluate flavour-specific effects. Compliance with exclusive nicotine pouch use was monitored through electronic product-use diaries completed by the participants. However, no objective measures were used to verify compliance, and therefore occasional use of tobacco-based snus or other nicotine products during the intervention period cannot be completely excluded. Furthermore, the effects observed in this study relate only to pre-existing lesions and may not be generalizable to individuals without prior tobacco or nicotine exposure. Histological analysis showed no significant differences in inflammatory cell presence or epithelial thickness between Visit 3 and Visit 5. These findings should, however, be interpreted with caution. Several biopsy samples were excluded from the analyses due to technical and alignment-related issues, reducing the number of evaluable specimens and thereby potentially limiting the ability to detect subtle tissue-level changes. Furthermore, inflammatory cell assessment was based on morphology-based quantification of total inflammatory cells in H&E-stained sections rather than immunohistochemical characterization of specific inflammatory cell populations. Consequently, more subtle changes in inflammatory cell composition or phenotype may have remained undetected despite the observed clinical improvement in lesion severity.

Analysis of saliva revealed that participants randomized to the dry formulation exhibited significantly increased IL-8 levels at Visit 5 compared with Visit 3. One limitation of the present study is that the exact cause of this increase remains unclear. The observed increase was not accompanied by significant changes in other major pro-inflammatory cytokines, suggesting that it may not reflect a broader inflammatory response. Another limitation is the relatively short duration of the study. While four weeks may not fully capture the long-term effects of non-tobacco-based nicotine pouch use, previous studies on snus-induced lesions have demonstrated that mucosal changes caused by smokeless tobacco are generally reversible within a relatively short period, often within 2–6 weeks after cessation or substitution of use [17, 32]. Therefore, a 4-week intervention period was considered sufficient to evaluate early clinical changes in lesion severity following product substitution.

Future studies including more detailed characterization of immune cell populations and local tissue expression of IL-8 are warranted to further clarify the biological mechanisms underlying lesion improvement and the observed cytokine changes.

## Conclusion

Within the limitations of this short-term study, substitution with nicotine pouches was associated with clinical improvement of pre-existing snus-induced lesions.

## Supplementary Information

Below is the link to the electronic supplementary material.

Supplementary Material 1

Supplementary File 2 Participant flow diagram illustrating screening, exclusions, randomization, allocation to treatment arms, study completion, and inclusion in the final analysis

High Resolution Image

## Data Availability

The datasets generated during and/or analyzed during the current study are available from the corresponding author on reasonable request.
